# Cytochrome P450 2E1 *Rsa*I/*Pst*I polymorphism and risk of esophageal cancer: A meta-analysis of 17 case-control studies

**DOI:** 10.3892/etm.2012.687

**Published:** 2012-08-28

**Authors:** WEI-DONG LENG, XIAN-TAO ZENG, YONG-JI CHEN, XIAO-LI DUAN, YU-MING NIU, RONG-PEI LONG, ZHI-XIAO LUO

**Affiliations:** 1 Department of Oral and Maxillofacial-Head and Neck Surgery, Centre of Stomatology and; 2 Department of Digestion, Taihe Hospital, Hubei University of Medicine, Shiyan, Hubei 442000;; 3 Department of Foreign Languages, Hubei University of Medicine, Shiyan, Hubei 442000, P.R. China

**Keywords:** cytochrome P450 2E1, polymorphism, esophageal cancer, mainland Chinese, meta-analysis

## Abstract

The aim of this study was to explore the cytochrome P450 2E1 (CYP2E1) *Rsa*I/*Pst*I polymorphism and risk of esophageal cancer (EC) in mainland Chinese populations. A systematic search of PubMed, EMBASE, Web of Science, CBM, CNKI and VIP databases for publications on the CYP2E1 *Rsa*I/*Pst*I polymorphism and risk of EC was performed. and the genotype data were analyzed in a meta-analysis. Odds ratios (ORs) with relevant 95% confidence intervals (CIs) were estimated to assess the association. Sensitivity analysis, test of heterogeneity and assessment of publication bias were performed. The search yielded 17 studies including 18 trails involving 1,663 cases and 2,603 controls. The meta-analyses showed a significant association between the CYP2E1 *Rsa*I/*Pst*I polymorphism and risk of EC in the mainland Chinese population (c2 vs. c1: OR=0.64; 95% CI, 0.50–0.81; P<0.001; c2/c2 vs. c1/c1: OR=0.73; 95% CI, 0.57–0.93; c2/c2 vs. c1/c1+c1/c2: OR=0.76; 95% CI, 0.60–0.96; P=0.02; c1/c2 vs. c1/c1: OR=0.54; 95% CI, 0.38–0.75; P<0.001; c1/c2+c2/c2 vs. c1/c1: OR=0.48; 95% CI, 0.34–0.70; P<0.001). An increased cancer risk in all genetic models was identified following stratification by ethnicity, source of controls and tumor type. In conclusion, in all genetic models, the association between the CYP2E1 *Rsa*I/*Pst*I polymorphism and risk of EC in the mainland Chinese population was significant. This meta-analysis suggests that the CYP2E1 *Rsa*I/*Pst*I polymorphism is a risk factor for EC, and the c2 allele is a factor that lowers the possibility of EC in the mainland Chinese population and this association did not change due to ethnic differences in genetic backgrounds and the environment.

## Introduction

Esophageal cancer (EC) ranks as the eighth most common malignancy and the sixth most common cancer-related cause of mortality worldwide, which is characterized by a high incidence and a striking worldwide geographic variation. In fact there exists a so-called ‘Asian esophageal cancer belt’, an area that stretches from the Caucasian mountains across Northern Iran, Afghanistan, Kazakhstan, Uzbekistan and Turkmenistan, into northern China, and China, Iran and Japan have the highest rate of EC in the world (1–3). These high incidence areas are associated with poverty or poverty-related diseases, and current evidence also reveals that the incidence of EC tends to decrease when wealth and accessibility to health care increases (4,5).

However, the cause and pathogenesis of EC remains unclear. Histopathologically, EC is classified into two main types: esophageal squamous cell carcinoma (ESCC) and esophageal adenocarcinoma (EAC). A higher incidence rate of EAC occurs in Western countries, while ESCC occurs more often in Oriental countries, particularly in Mainland China (1,2,6). The possible risk factors for developing EC have been reported to be alcohol consumption, smoking, coffee consumption, low socioeconomic status, poor oral health, hot drinks, high consumption of nitrosamines, diet deficient in antioxidants and other environmental factors (1–3,5,7,8). Recent studies reveal that genetic alterations are also considered as new risk factors for EC (9,10).

Numerous studies have shown that the cytochrome P450 superfamily are catalyzing enzymes for carcinogens. Cytochrome P450 2E1 (CYP2E1), a member of the CYP450 superfamily and an ethanol-inducible enzyme, is involved in the metabolic activation of numerous low molecular weight compounds, including N-nitrosamines, aniline, vinyl chloride and urethane (11–14). *Rsa*I/*Pst*I polymorphisms in the promoter gene region are believed to affect the transcriptional activity of the gene, and occur as a wild-type homozygous genotype (c1/c1), a heterozygous genotype (c1/c2) and a variant homozygous rare genotype (c2/c2), and the frequency of the variant c1 allele was observed to be much higher in patients with esophageal diseases than that of healthy individuals (15–17).

The first study on the association between EC and the CYP2E1 *Rsa*I/*Pst*I polymorphism was conducted in 1996, but the result failed to reveal a significant association. The second study in Japan also failed to identify a significant difference between healthy controls and patients with EC (18). However, certain subsequent studies on Chinese individuals indicated that a significant association exists between the CYP2E1 *Rsa*I/*Pst*I polymorphism and the risk of EC, yet certain studies revealed different or even contradictory findings (16,17,19–33).

A previous meta-analysis was carried out concerning EC and the CYP2E1 *Rsa*I/*Pst*I polymorphism, of which 11 published studies demonstrated that the CYP2E1 *Rsa*I/*Pst*I c2 allele may be a protective factor for EC among Asian populations (34). In the included 7 Chinese studies, the authors only searched PubMed and did not categorize subgroups according to the source of controls, which makes their studies unsuitable for reference for mainland Chinese populations. Therefore, we conducted a meta-analysis of case-control studies in order to review and summarize the epidemiological evidence, and aimed to precisely detect the correlation between the CYP2E1 *Rsa*I/*Pst*I polymorphism and the risk of EC among Chinese individuals.

## Materials and methods

### Literature search

Strictly following the proposed Preferred Reporting Items for Systematic Reviews and Meta-Analyses (PRISMA) (35) guidelines, our study aims to report the present review and meta-analysis systematically. Initially, we identified published and unpublished studies which tested the association between the CYP2E1 *Rsa*I/*Pst*I polymorphism and risk of EC by searching the following databases from their creation until January 10th, 2012: PubMed, EMBASE, Web of Science, the Chinese Biological Medicine Database (CBM), China National Knowledge Infrastructure (CNKI) and the Chinese scientific periodical database of VIP information (VIP). Terms used in the search were as follows: i) esophag^*^ and oesophag^*^; ii) cancer, carcinoma, adenocarcinoma, neoplasm, neoplasia and neoplastic; iii) cytochrome p450 2E1, cytochrome p450II1, CYP2E1, CYPIIE1; iv) polymorphism; v) China and Chinese, without restrictions. In addition, we also reviewed the reference lists of retrieved manuscripts and recent reviews.

### Study selection

We included any study that met with the following criteria: i) case-control study design; ii) association between CYP2E1 *Rsa*I/*Pst*I polymorphism and risk of EC was investigated; iii) diagnosis of ESCC and EAC were either histologically, pathologically or cytologically confirmed; and iv) the odds ratios (ORs) and the corresponding 95% confidence intervals (CIs), or the number of events required to calculate them were reported. Two investigators independently evaluated the eligibility of all studies retrieved from the databases on the basis of the predetermined selection criteria. Disagreements were resolved by discussion or in consultation with a third investigator.

### Data extraction

Two reviewers independently extracted data for the study characteristics by using a standardized data-collection form. Data were recorded as follows: first author’s last name, year of publication, place of origin, study period and duration of follow-up, characteristics of cancer cases, source of controls, matching criteria, number of cases and controls, number of different genotypes in cases and controls, Hardy-Weinberg equilibrium (HWE) and minor allele frequency in controls. Any disagreements were resolved by consensus.

### Statistical analysis

We computed a pooled OR and 95% CI for the risk allele using the Comprehensive Meta Analysis software (version 2.1) to generate forest plots to determine whether there was a statistical association between cases and controls and to assess heterogeneity of the included studies. HWE was tested by a Chi-square test at a significance level of P<0.05. Heterogeneity was quantifiably evaluated using the Chi-square based Cochran’s Q statistic (36) and the I^2^ statistic, which yields results ranging from 0 to 100% (I^2^=0–25%, no heterogeneity; I^2^=25–50%, moderate heterogeneity; I^2^=50–75%, large heterogeneity; I^2^=75–100%, extreme heterogeneity) (37). If heterogeneity existed, the random-effects model was used, otherwise, the fixed-effects model was used. In addition, we investigated the influence of a single study to the overall risk estimate by removing each study in turn to test the robustness of the main results. Subgroup analysis was also conducted if significant heterogeneity was identified (such as Han individuals vs. ethnic minorities). If possible, potential publication bias was assessed by visual inspection of the funnel plots of the primary outcome (38).

## Results

### Identification of eligible studies

**Figure 1:**
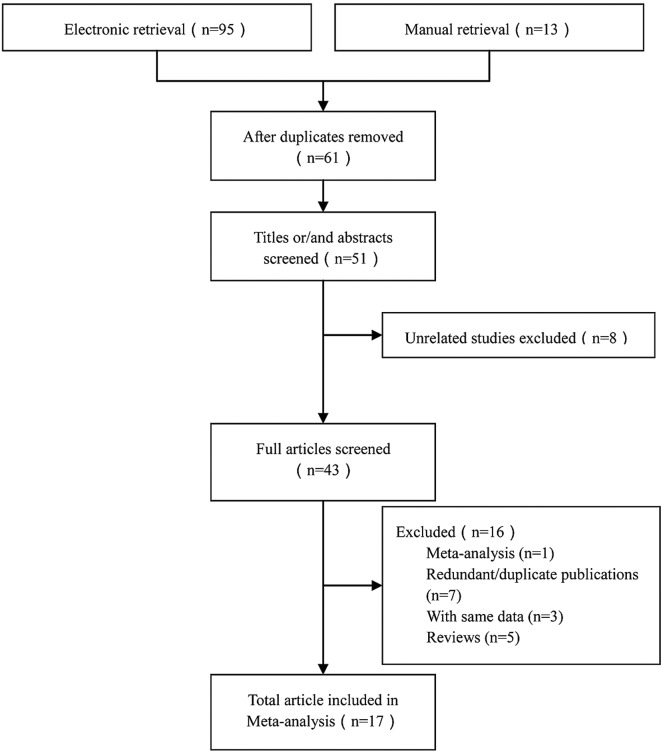
Summary of the study selection process.

Of the 108 records retrieved initially, 17 studies including 18 trails were identified for the association between the CYP2E1 *Rsa*I/*Pst*I polymorphism and risk of EC, including a total of 1,663 cases and 2,603 controls (16,17,19–33). A flow chart for the study selection process is presented in Fig. 1. Duplicates (the same study searched from different databases) were excluded using the Endnote X3 software.

### Characteristics of the studies

The detailed characteristics of the included studies are summarized in Table I. Of these studies, 7 were published in English (16,17,19,22,28–30) and 7 in Chinese (21,23–25,27,31,33), 2 were doctoral dissertations (20,32) and 1 was a master’s thesis both in English and Chinese (26). The sample sizes ranged from 45 to 480. All of the cases were histologically, pathologically or cytologically confirmed as EC. Of the cases, 6 studies clearly confirmed the presence of ESCC (17,19,28,29,31,32), while one had both ESCC and EAC (16). Controls were mainly healthy populations and matched according to age, gender or were cancer-free tissues. Of the controls 6 were hospital-based (HB) (20,23–25,27,31), 10 were population-based (PB) (16,17,19,21,22,26,28,29,32,33) and one was both (30). There were two groups of Kazakhs (Xinjiang Province) (19,30) and 16 groups of Han individuals (16,17,20–29,31–33). The genotypes were analyzed by polymerase chain reaction-restriction fragment length polymorphism (PCR-RFLP); genotype distributions in the controls of all studies were in accordance with HWE, with the exception of 4 studies (17,19,23,29).

### Meta-analyses

The main results of the heterogeneity test and meta-analysis are listed in Table II.

**Figure 2:**
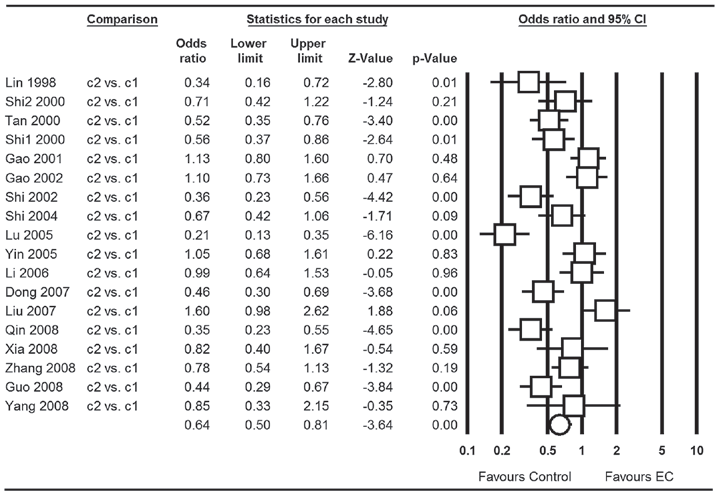
Forest plot of EC associated with CYP2E1 *Rsa*I/*Pst*I for the c2 allele compared with the c1 allele in total. CI, confidence interval; EC, esophageal cancer.

**Figure 3:**
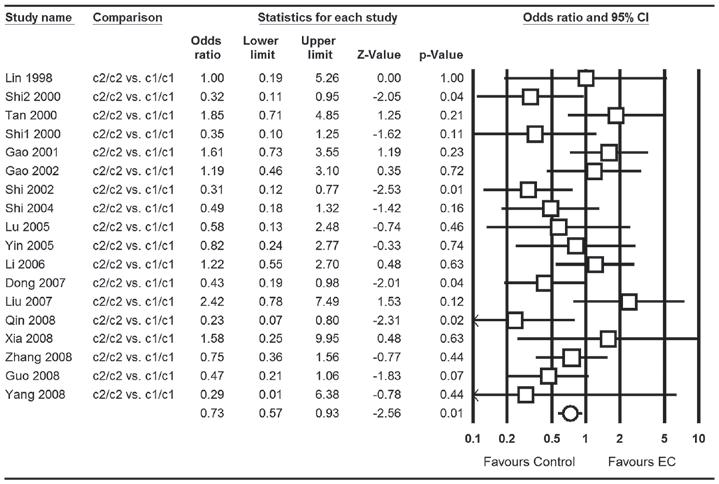
Forest plot of EC associated with CYP2E1 *Rsa*I/*Pst*I for the c2/c2 genotype compared with the c1/c1 genotype in total. CI, confidence interval; EC, esophageal cancer.

**Figure 4:**
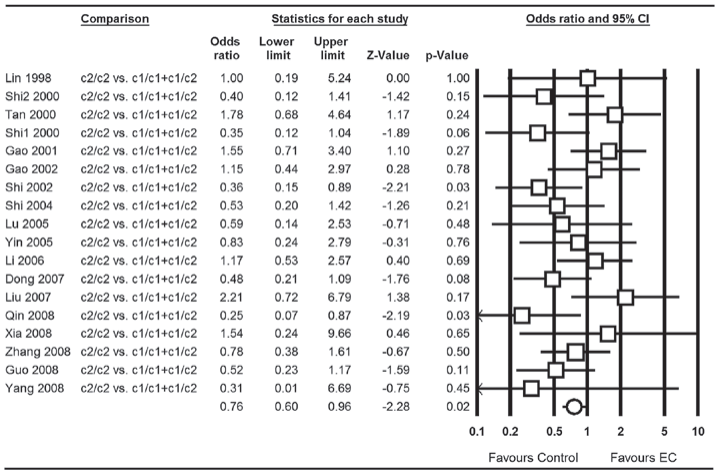
Forest plot of EC associated with CYP2E1 *Rsa*I/*Pst*I for the c2/c2 genotype compared with the c1/c1+c1/c2 genotype in total. CI, confidence interval; EC, esophageal cancer.

**Figure 5:**
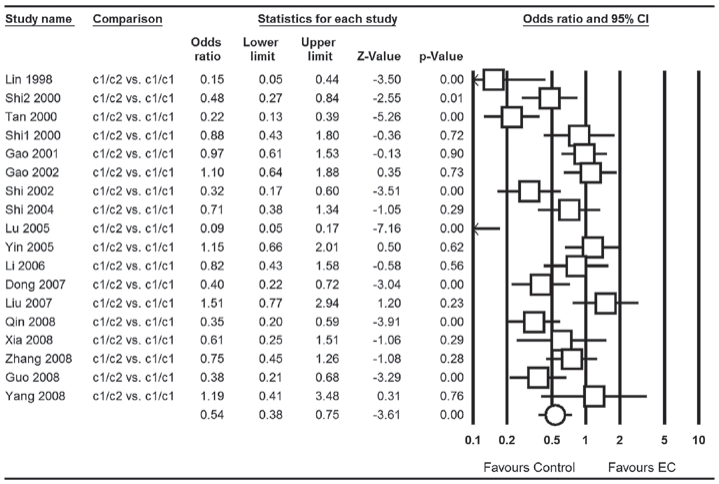
Forest plot of EC associated with CYP2E1 *Rsa*I/*Pst*I for the c1/c2 genotype compared with the c1/c1 genotype in total. CI, confidence interval; EC, esophageal cancer.

**Figure 6:**
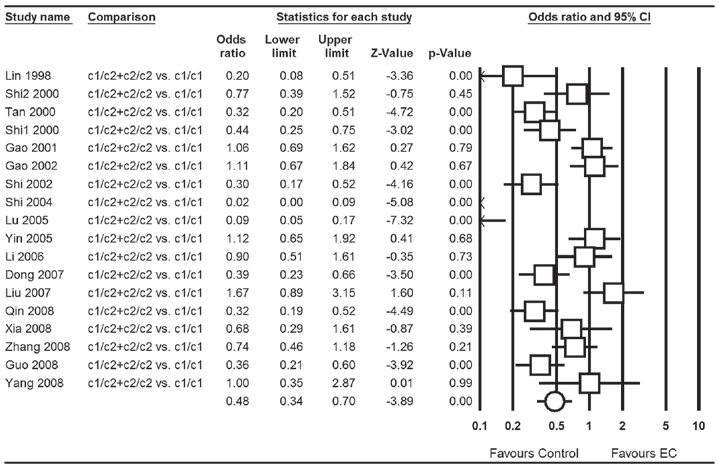
Forest plot of EC associated with CYP2E1 *Rsa*I/*Pst*I for the c1/c2+c2/c2 genotype compared with the c1/c1 genotype in total. CI, confidence interval; EC, esophageal cancer.

Our meta-analyses gave a significant association of the CYP2E1 *Rsa*I/*Pst*I polymorphism with EC risk [for the allele contrast c2 vs. c1: OR=0.64; 95% CI, 0.50–0.81; P<0.001 (Fig. 2); for c2/c2 vs. c1/c1: OR=0.73; 95% CI, 0.57–0.93; P=0.01 (Fig. 3); for c2/c2 vs. c1/c1+c1/c2: OR=0.76; 95% CI, 0.60–0.96; P=0.02 (Fig. 4); for c1/c2 vs. c1/c1: OR=0.54; 95% CI, 0.38–0.75; P<0.001 (Fig. 5); for the dominant model c1/c2+c2/c2 vs. c1/c1: OR=0.48; 95% CI, 0.34–0.70; P<0.001 (Fig. 6)] in total populations.

When stratified by ethnicity, all genetic models also produced statistically significant results. When studies were stratified for control source, an association was detected for all genetic models, with the exception of PB in c2/c2 vs. c1/c1 and c2/c2 vs. c1/c1+c1/c2 (both P>0.05). In the stratified analysis by tumor type, the results were similar to the control source; the ESCC type showed no association in c2/c2 vs. c1/c1 and c2/c2 vs. c1/c1+c1/c2 (both P>0.05).

### Sensitivity analysis

**Figure 7:**
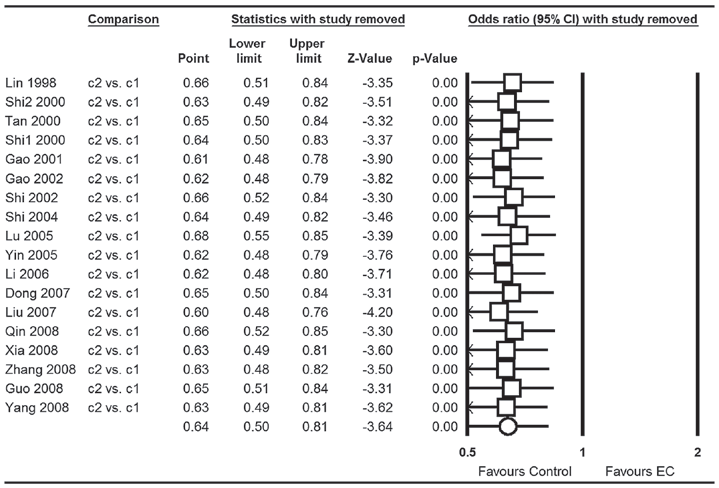
Sensitivity analysis based on c2 vs. c1; a single study was removed each turn. CI, confidence interval; EC, esophageal cancer.

The majority of studies indicated that the frequency distributions of genotypes in the controls were in accordance with HWE, whereas deviations from HWE were observed in 4 studies of the *Pst*I/*Rsa*I polymorphism (all P<0.05) (17,19,23,29). However, the corresponding pooled ORs were not substantially altered whether or not these studies were included (Table III). In addition, sensitivity analysis indicated that no single study influenced the pooled OR qualitatively and this suggests the stability of the result (c2 vs. c1 for example; Fig. 7).

### Publication bias

**Figure 8:**
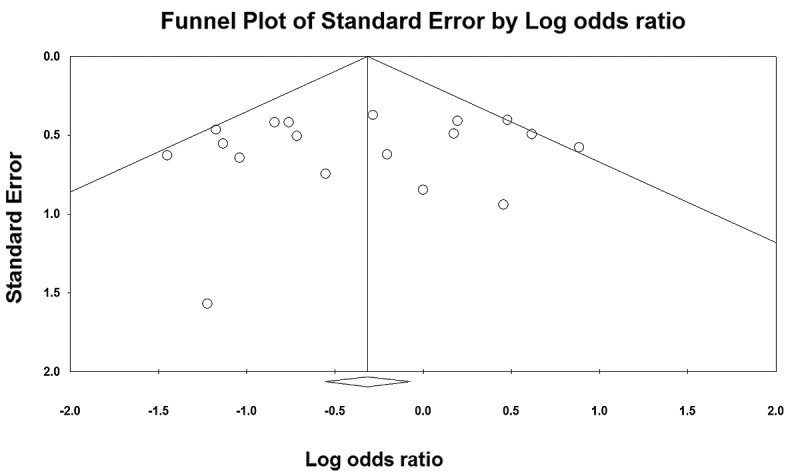
Funnel plot based on the c2/c2 vs. c1/c1+c1/c2 genetic model.

Funnel plot based on c2/c2 vs. c1/c1 (the genetic model was pooled using a fixed-effects model) was chosen to assess publication bias. The symmetrical shape of the funnel plots (Fig. 8) implied that slight bias of the studies occurred.

## Discussion

EC is an increasingly common cancer with a poor prognosis, which is likely to be caused by multi-factors, including environmental risk and genetic factors (3,7–9). In recent years, environmental and genetic susceptivity, and their interactions, were used to evaluate the risks of EC, but the results were inconsistent and this difference may be due to geographical distribution. Environmental risk factors were once regarded as the major cause of EC, but an epidemiological study on immigrants moving to Changzhi City in Shanxi Province after a century of residing in Linzhou City in Henan Province (>200 km apart and the environment is different), observed that the detection rate of ESCC in the immigrant population was similar to that of the residents in Linzhou City. It indicated that changes in environment and time do not affect the incidence rate of ESCC, since genetic factors play an important role (39).

Unlike other factors, the positive association between family history of EC risk is consistent with previous studies, which suggests a genetic susceptibility in EC pathogenesis (40–50). A meta-analysis in 2003 involved 13 studies to evaluate risk factors of EC in China and revealed that family history was a significant factor (OR=4.0; 95% CI, 2.29–6.99) (51). The majority of studies concluded that this is caused by various hereditary susceptibilities to gene-related tumors. As for genetic susceptibility, it has been reported that gene polymorphisms of metholenetetrahydrofolate reductase (MTHFR) (52), cytochrome P450 1A1 (CYP1A1) (52), glutathione S-transferase M1 (GSTM1) (52), GSTT1 (53), CYP2A6 (54), CYP2E1 (34), human 8-oxoguanine glycosylase 1 (hOGG1) (55), X-ray repair cross-complementing group 1 (XRCC1) (56), xeroderma pigmentosum group D (XPD) (56), p53 (57) and others, are correlated with EC.

The CYP2E1 gene that encodes the CYP2E1 enzyme has been mapped to chromosome 10q24.3-qter, and is an important member of the cytochrome P450 superfamily. CYP2E1 is a naturally ethanol-inducible enzyme mainly involved in the metabolic activation of low molecular weight compounds, such as N-nitrosamines and in alcohol metabolism (11–14). The *Rsa*I/*Pst*I polymorphisms in the promoter gene region are reported to affect the transcriptional activity of the gene. Numerous large-sample and unbiased epidemiological studies of CYP2E1 *Rsa*I/*Pst*I polymorphisms could confirm it as a predisposition gene for EC risk, particularly in China (15–33). Molecular biological studies have also demonstrated that the rare allele of the *Rsa*I/*Pst*I polymorphism in the CYP2E1 gene is associated with increased transcriptional activity (58), which may play an important role in EC development.

Our meta-analysis summarized all the available data on the association between the CYP2E1 *Rsa*I/*Pst*I polymorphism and EC risk, including a total of 4,266 subjects. The results clearly suggested that there was a significant association between the CYP2E1 *Rsa*I/*Pst*I polymorphism and EC susceptibility. The *Rsa*I/*Pst*I c2 allele is a factor which lowers the possibility of EC, which may change and increase the ability to activate mutagens and carcinogens (OR=0.64; 95% CI, 0.50–0.81).

When subgroups were analyzed by ethnicity, the c2 allele was considered as a decreased risk factor in both Han and Kazakh subgroups, suggesting the ethnic differences in genetic backgrounds and the environment they lived in was non-related factor, which was in accordance with Wang *et al* (39).

In mainland China, ESCC is one of the most common malignancies, and has a great geographic variation of occurrence; the Northwest of China shows an exceptionally high occurrence (2). However, the meta-analysis of ESCC subgroup failed to identify any significant association in c2/c2 vs. c1/c1 (OR=0.94; 95% CI, 0.61–1.46; P=0.80) and c2/c2 vs. c1/c1+c1/c2 (OR=0.96; 95% CI, 0.62–1.49, P=0.87) genetic models. This phenomenon could have resulted since the included studies did not indicated the tumor type were all ESCC, with the exception of Lin *et al* (16) (both ESCC and EAC). Thus, future studies should clearly report the cancer type included.

Similar results also appeared in the PB controls in the c2/c2 vs. c1/c1 (OR=1.02; 95% CI, 0.76–1.38; P=0.89) and c2/c2 vs. c1/c1+c1/c2 (OR=1.03; 95% CI, 0.76–1.38; P=0.54) genetic models, and this could be since the HB studies have certain biases for such controls and may only represent a sample of an ill-defined reference population, and may not be representative of the general population; or it may be that numerous subjects in the PB controls were susceptible individuals. Therefore, the use of proper and representative PB control subjects is important to reduce biases in such genetic studies.

The sensitivity of the frequency distributions of genotypes in the controls were inconsistent with HWE, and the stability of results by deleting one study each time suggested that they were not substantially altered and the results were stable. Hence, the heterogeneity of the studies did not substantially lower the statistical validity of the study and the CYP2E1 *Rsa*I/*Pst*I polymorphism is clearly associated with EC risk in Chinese individuals.

Compared with the previous meta-analysis (34), this meta-analysis grouped subgroups with more accuracy than before, and contained more studies and a more accurate association estimation. Certain limitations of our meta-analysis must be acknowledged. Firstly, heterogeneity among the studies, resulting from different defined controls or other factors, may influence the results of this analysis. In certain studies, the controls were selected randomly from a healthy or normal population, but in other studies controls were selected from HB cancer-free patients. In addition, the matching criteria of the control group differed in age and gender. The variant risks (eg. gender, living habits) of EC in these different populations may affect the results. Secondly, it is well-known that a single gene has only a moderate effect on EC development. The combinations of certain genotypes may be the more discriminating factor than a single locus genotype. In our meta-analysis, just as the previous one, the connection between disequilibrium and haplotype analysis was not performed. Thirdly, although primary studies had adjusted for covariates, the ORs of this meta-analysis were obtained without correction, while a more precise analysis should be performed if individual data were available, which would allow for the adjustment by the covariates, including age, gender, ethnicity, smoking and other factors. Fourthly, the sample size was still relatively small; thus, we could not fully assess the effects. Finally, a potential limitation of any meta-analysis is the ‘file-drawer’ effect, in which studies with negative results may remain unpublished, and this may bias the literature toward positive findings. Although we endeavored to search for unpublished studies and the funnel plots also did not detect obvious publication bias, we still cannot confirm all the relevant studies were included.

In conclusion, evidence from the included studies suggests that the CYP2E1 *Rsa*I/*Pst*I polymorphism plays an important role in EC development for mainland Chinese individuals. It also suggests that the c2 allele significantly decreases the susceptibility to EC. For future study, studies with larger sample sizes, stricter selection of patients, well-matched PB controls and clearly reported cancer types are required. In addition, the potential gene-gene and gene-environment interactions of the CYP2E1 *Rsa*I/*Pst*I polymorphism and EC should be further investigated.

## Figures and Tables

**Table I:** Characteristics of the studies included in this meta-analysis.

Author (Refs.)	Site	Group	Source	Sample	Genotype			Genotyping method	P-value (HWE) for controls	Adjustment
					c1/c1	c1/c2	c2/c2			
Lin *et al* (16)	Linxian County, Henan Province	T	ESCC and EAC	45	36	6	3	PCR-RFLP	0.345	Age, gender
		C	PB	45	20	22	3			
Tan *et al* (17)	Linxian County, Henan Province	T	ESCC	150	107	31	12	PCR-RFLP	0.009	Age, gender, smoking
		C	PB	150	66	77	7			
Shi (20)	Linzhou City, Henan Province	T	EC	116	74	37	5	PCR-RFLP	0.922	Age, gender, smoking, fermented vegetable
		C	HB	106	46	48	12			
	Xi’an City, Shaanxi Province	T	EC	71	39	28	4	PCR-RFLP	0.368	
		C	HB	62	30	24	8			
Gao *et al* (21)	Huai’an City, Jiangsu Province	T	EC	144	83	44	13	PCR-RFLP	0.525	Age, gender, tea, smoking, alcohol
		C	PB	233	140	75	14			
Shi *et al* (23)	Wuhan City, Hubei Province	T	EC	98	72	19	7	PCR-RFLP	0.039	Age, gender
		C	HB	120	54	45	21			
Gao *et al* (22)	Huai’an City, Jiangsu Province	T	EC	93	55	31	7	PCR-RFLP	0.20	Age, gender, smoking, drinking, dietary habits
		C	PB	196	121	62	13			
Shi *et al* (24)	Nanjing City, Jiangsu Province	T	EC	78	48	24	6	PCR-RFLP	0.141	Age, gender, smoking, alcohol
		C	HB	118	60	42	16			
Yin *et al* (25)	Huai’an City, Jiangsu Province	T	EC	106	52	49	5	PCR-RFLP	0.411	Age, gender
		C	HB	106	55	45	6			
Lu *et al* (19)	Kazakh, Xinjiang Autonomous Region	T	ESCC	104	81	20	3	PCR-RFLP	<0.001	Age, gender
		C	PB	104	25	74	5			
Li (26)	Taiyuan City, Shanxi Province	T	EC	60	32	18	10	PCR-RFLP	0.781	Age, gender, smoking, drinking habits
		C	PB	199	101	69	29			
Liu *et al* (28*)*	Huai’an City, Jiangsu Province	T	ESCC	77	34	33	10	PCR-RFLP	0.91	Age, gender
		C	PB	79	45	29	5			
Dong *et al* (27)	Gansu Province	T	EC	120	84	26	10	PCR-RFLP	0.428	Age, gender
		C	HB	120	57	44	19			
Xia *et al* (31)	Jingjiang City, Jiangsu Province	T	ESCC	45	30	12	3	PCR-RFLP	0.708	Not reported
		C	HB	45	26	17	2			
Qin *et al* (30)	Kazakh, Xinjiang Autonomous Region	T	EC	120	94	23	3	PCR-RFLP	0.29	Age, gender, dietary habits
		C	PB and HB	240	128	90	22			
Guo *et al* (29)	Lanzhou City, Gansu Province	T	ESCC	80	57	16	7	PCR-RFLP	<0.001	Age, gender
		C	PB	480	225	180	75			
Yang (32)	Feicheng City, Shandong Province	T	ESCC	27	19	8	0	PCR-RFLP	0.442	Age, gender, alcohol, education, smoking
		C	PB	44	31	11	2			
Zhang and Wu (33)	Gansu Province	T	EC	129	75	40	14	PCR-RFLP	0.443	Age, gender
		C	PB	156	70	56	21			

Characteristics of studies included in this meta-analysis. T, case subjects; C, control subjects; PB, population-based; HB, hospital-based; ESCC, esophageal squamous cell carcinoma; EAC, esophageal adenocarcinoma; EC, esophageal cancer; HWE, Hardy-Weinburg equilibrium; PCR-RFLP, polymerase chain reaction-restriction fragment length polymorphism.

**Table II:** Main results of the heterogeneity test and subgroups meta-analyses.

			Heterogeneity		Meta-analyses	
Genetic model	Study or subgroup		P-value	I^2^ (%)	OR (95% CI)	P-value
c2 vs. c1	Total		<0.001	80	0.64 (0.50–0.81)	0.0003
	Ethnicity	Han	<0.001	72	0.71 (0.57–0.89)	0.002
		Kazakh	0.12	58	0.28 (0.17–0.46)	<0.001
	Source of control	PB	<0.001	85	0.65 (0.45–0.53)	0.02
		HB	0.02	59	0.62 (0.46–0.82)	0.0007
		Both	Single study		0.35 (0.23–0.55)	<0.001
	Tumor type	EC	<0.001	76	0.69 (0.53–0.89)	0.005
		ESCC	<0.001	84	0.56 (0.33–0.93)	0.03
c2/c2 vs. c1/c1	Total		0.03	42	0.70 (0.56–0.89)	0.003
	Ethnicity	Han	0.04	42	0.75 (0.59–0.95)	0.02
		Kazakh	0.35	0	0.32 (0.13–0.80)	0.01
	Source of control	PB	0.30	16	1.02 (0.76–1.38)	0.89
		HB	0.69	0	0.44 (0.30–0.66)	<0.001
		Both	Single study		0.23 (0.07–0.80)	0.02
	Tumor type	EC	0.05	46	0.63 (0.48–0.82)	0.0008
		ESCC	0.19	31	0.94 (0.61–1.46)	0.80
c1/c2 vs. c1/c1	Total		<0.001	81	0.54 (0.38–0.75)	0.0003
	Ethnicity	Han	<0.001	72	0.62 (0.46–0.84)	0.002
		Kazakh	0.002	90	0.18 (0.05–0.68)	0.01
	Source of control	PB	<0.001	88	0.52 (0.30–0.92)	0.02
		HB	0.05	53	0.59 (0.42–0.85)	0.004
		Both	Single study		0.35 (0.20–0.59)	<0.001
	Tumor type	EC	0.004	62	0.66 (0.50–0.88)	0.004
		ESCC	<0.001	87	0.38 (0.18–0.82)	0.01
c2/c2 vs. c1/c1+c1/c2	Total		0.12	29	0.73 (0.58–0.92)	0.008
	Ethnicity	Han	0.15	27	0.78 (0.61–0.99)	0.04
		Kazakh	0.38	0	0.34 (0.13–0.85)	0.02
	Source of control	PB	0.47	0	1.03 (0.76–1.38)	0.54
		HB	0.8	0	0.49 (0.33–0.72)	0.0003
		Both	Single study		0.25 (0.07–0.87)	0.03
	Tumor type	EC	0.14	33	0.66 (0.50–0.87)	0.003
		ESCC	0.31	16	0.96 (0.62–1.49)	0.87
c1/c2+c2/c2 vs. c1/c1	Total		<0.001	85	0.48 (0.34–0.70)	0.0001
	Ethnicity	Han	<0.001	81	0.56 (0.40–0.79)	0.001
		Kazakh	0.003	85	0.17 (0.05–0.59)	0.009
	Source of control	PB	<0.001	88	0.56 (0.33–0.95)	0.03
		HB	<0.001	82	0.42 (0.23–0.75)	0.004
		Both	Single study		0.32 (0.19–0.52)	<0.001
	Tumor type	EC	<0.001	82	0.55 (0.37–0.82)	0.003
		ESCC	<0.001	87	0.41 (0.21–0.85)	0.02

The main results of the heterogeneity test and meta-analysis. OR, odds ratio; CI, confidence interval; EC, esophageal cancer; ESCC, esophageal squamous cell carcinoma; PB, population-based; HB, hospital-based.

**Table III:** Sensitivity analysis after elimination of the studies where frequency distributions of genotypes in the controls were inconsistent with HWE.

		Heterogeneity		Meta-analyses		
Genetic model	Subgroup	P-value	I^2^ (%)	OR (95% CI)	P-value	Model
c2 vs. c1	Total	<0.001	80	0.64 (0.50, 0.81)	0.0003	Random
	AE	<0.001	71	0.76 (0.60, 0.96)	0.02	Random
c2/c2 vs. c1/c1	Total	0.03	42	0.70 (0.56, 0.89)	0.003	Fixed
	AE	0.08	37	0.75 (0.57, 0.98)	0.03	Fixed
c1/c2 vs. c1/c1	Total	<0.001	81	0.54 (0.38, 0.75)	0.0003	Random
	AE	0.001	62	0.71 (0.54, 0.93)	0.01	Random
c2/c2 vs. c1/c1+c1/c2	Total	0.12	29	0.73 (0.58, 0.92)	0.008	Fixed
	AE	0.19	24	0.77 (0.59, 1.00)	0.05	Fixed
c1/c2+c2/c2 vs. c1/c1	Total	<0.001	85	0.48 (0.34, 0.70)	0.0001	Random
	AE	<0.001	80	0.62 (0.43, 0.89)	0.01	Random

Sensitivity analysis after elimination of the studies for which the frequency distributions of genotypes in the controls were inconsistent with HWE. AE, after elimination; HWE, Hardy-Weinburg equilibrium; OR, odds ratio; CI, confidence interval.
